# Identification of indels in next-generation sequencing data

**DOI:** 10.1186/s12859-015-0483-6

**Published:** 2015-02-13

**Authors:** Aakrosh Ratan, Thomas L Olson, Thomas P Loughran, Webb Miller

**Affiliations:** Center for Comparative Genomics and Bioinformatics, Pennsylvania State University, 506, Wartik Laboratory, University Park, PA 16802 USA; Department of Medicine, Hematology and Oncology, and the University of Virginia Cancer Center, University of Virginia, Charlottesville, VA 22908 USA; Department of Public Health Sciences and Center for Public Health Genomics, University of Virginia, Charlottesville, VA 22908 USA

**Keywords:** Indels, Variants, Sequencing analysis

## Abstract

**Background:**

The discovery and mapping of genomic variants is an essential step in most analysis done using sequencing reads. There are a number of mature software packages and associated pipelines that can identify single nucleotide polymorphisms (SNPs) with a high degree of concordance. However, the same cannot be said for tools that are used to identify the other types of variants. Indels represent the second most frequent class of variants in the human genome, after single nucleotide polymorphisms. The reliable detection of indels is still a challenging problem, especially for variants that are longer than a few bases.

**Results:**

We have developed a set of algorithms and heuristics collectively called indelMINER to identify indels from whole genome resequencing datasets using paired-end reads. indelMINER uses a split-read approach to identify the precise breakpoints for indels of size less than a user specified threshold, and supplements that with a paired-end approach to identify larger variants that are frequently missed with the split-read approach. We use simulated and real datasets to show that an implementation of the algorithm performs favorably when compared to several existing tools.

**Conclusions:**

indelMINER can be used effectively to identify indels in whole-genome resequencing projects. The output is provided in the VCF format along with additional information about the variant, including information about its presence or absence in another sample. The source code and documentation for indelMINER can be freely downloaded from www.bx.psu.edu/miller_lab/indelMINER.tar.gz.

**Electronic supplementary material:**

The online version of this article (doi:10.1186/s12859-015-0483-6) contains supplementary material, which is available to authorized users.

## Background

Genetic differences between individuals are encoded as local changes consisting of substitutions and small indels that alter a few base pairs, and large-scale changes that consist of larger indels, rearrangements and copy number variations. Whole genome sequencing using NGS technologies offers a unique opportunity to study these variations and enable a better understanding of genome function and diversity. There are a number of mature software packages and associated pipelines that can identify single nucleotide polymorphisms (SNPs) with a high degree of concordance [1]. However, the same cannot be said for tools that are used to identify the other sources of variation.

Indels are the most common structural variant that contribute to pathogenesis of disease [2], gene expression and functionality. Current approaches to identify indels include de-novo assembly of unaligned reads [3], read splitting [4,5], depth of coverage analysis [6] and analysis of insert size inconsistencies. Each of these approaches has their own strengths and weaknesses. For example, even though de-novo assembly offers the best opportunity to accurately call these variants, assembly with short reads is a challenging problem that requires significant computational resources. Similarly split-read approaches perform with a high degree of accuracy for short and medium sized indels, but the false-negative rate increases significantly with increase in size of the variations. Paired-end read and depth of coverage approaches frequently miss small indels, and are unable to predict the breakpoints accurately. We believe a hybrid strategy that integrates the information using more than one of the above approaches is required to identify these indels with a high degree of sensitivity and specificity.

Here we present indelMINER, a method that uses a combination of split-read and paired-end approaches to identify the breakpoints of insertions and deletions. The identified indels can be annotated with additional information such as the depth of coverage across the predicted breakpoints, and the list can be subsequently filtered to generate a high quality subset of variants. In addition to identification of indels, indelMINER can also be used to investigate the absence or presence of support for a set of indels in another sample. This is valuable in investigation of normal/tumor pairs as well in cases where several individuals of a family are sequenced to identify de-novo changes in the proband, and a novel feature of indelMINER. We present the results of using indelMINER on simulated data as well as real data from the individual NA18507 and a cancer genome dataset. We compare the performance and results of indelMINER to previously published results from several other similar tools.

## Results

### Simulated dataset

In order to calculate the sensitivity and specificity of our method, and compare it to that of a few other popular tools, we implanted 3,723 known homozygous deletions, and 3,777 known homozygous insertions [7] into chromosome 22 of the human genome. 100 bp long paired-end (average insert distance 500 bps, s.d. 30 bps) Illumina reads were simulated from this modified sequence using pIRS [8], such that each nucleotide on the reference was covered 20 times on an average. The reads were mapped to the human reference chromosome 22 using BWA [9] version 0.5.9, with the default parameters. The resulting BAM file was sorted based on the chromosomal coordinates, and the reads were realigned around putative indels using IndelRealigner tool from the GATK suite [10].

We ran SAMtools [11], PINDEL [4], PRISM [5] and indelMINER on this dataset and the results are summarized in Table 1. The indels identified by the tools were compared to the true set, and in case of deletions a call was marked as validated if there was a reciprocal overlap with at least half of the actual deletion. The details of the arguments and parameters used for this experiment are detailed in the Additional file 1. SAMtools exhibits the lowest false-positive rate for this dataset (2.65%), but its false-negative rate is significantly higher when compared to the other software. Out of the remaining tools, indelMINER exhibits the lowest false-positive rate (3.57% compared to 4.06% for PRISM and 4.53% for PINDEL) as well a false-negative rate that is significantly lower than PINDEL (10.54% for indelMINER, 15.59% for PINDEL), and comparable to that of PRISM (10.46%).

**Table 1:** **Comparison of SAMtools, PINDEL, PRISM and indelMINER on simulated dataset with 3,723 deletions and 3,777 insertions**

**SV caller**	**Observed indels**	**False-positives**	**False-negatives**
SAMtools	6,491	172 (2.65%)	1181
PINDEL	7,239	328 (4.53%)	589
PRISM	7,406	301 (4.06%)	395
indelMINER	7,365	263 (3.57%)	398

### Real dataset

We used about 28-fold data corresponding to the Yoruban HapMap individual NA18507 (Accession: SRX016231) to evaluate indelMINER on real data. The same sample has been characterized in multiple studies [4,5,12,13] and sequenced using multiple platforms [14,15], making it an ideal test case to compare the results of indelMINER. We downloaded the fastq reads for the HapMap individual from the Short read archive (Accession: SRX016231). These 101 bp reads were generated using the standard Illumina paired-end library protocol, with an average insert length of about 500 bps. We aligned these reads to the hg19 reference sequence using BWA version 0.5.9 with the default parameters except -q 15, which was used to trim the low quality segment of the read down to 35 bps at the 3’ end. The reads around putative indels were realigned using GATK IndelRealigner, followed by use of MarkDuplicates (http://picard.sourceforge.net) to flag the potential PCR duplicates. The resulting BAM file was used to identify indels using SAMtools, PINDEL, PRISM and indelMINER (See Additional file 1).

For the NA18507 genome, indelMINER detected 643,636 indels (347,590 deletions and 296,046 insertions). Additional file 1: Figure S1 shows the length distribution of the identified indels and Additional file 1: Figure S2 shows their distribution across the human chromosomes, which correlates well to the amount of DNA present in the chromosomes. 313 of the identified indels overlap with the protein coding exons corresponding to the set of RefSeq [16] genes. 44.81% of these coding indels are of lengths that are a multiple of 3. This is in close concordance with previous studies [17,18] that have reported that in-frame indels should constitute about 50%-60% of all coding indels. 412,001 (64.01%) of these indels identified using indelMINER were also found in dbSNP version 137 and 454,120 (70.55%) of them were found in the Database of Genomic Variants (DGV). 220,434 (34.25%) of the variants were also identified in the Phase 1 release 3 of the 1000 genomes project in African samples.

**Figure 1:**
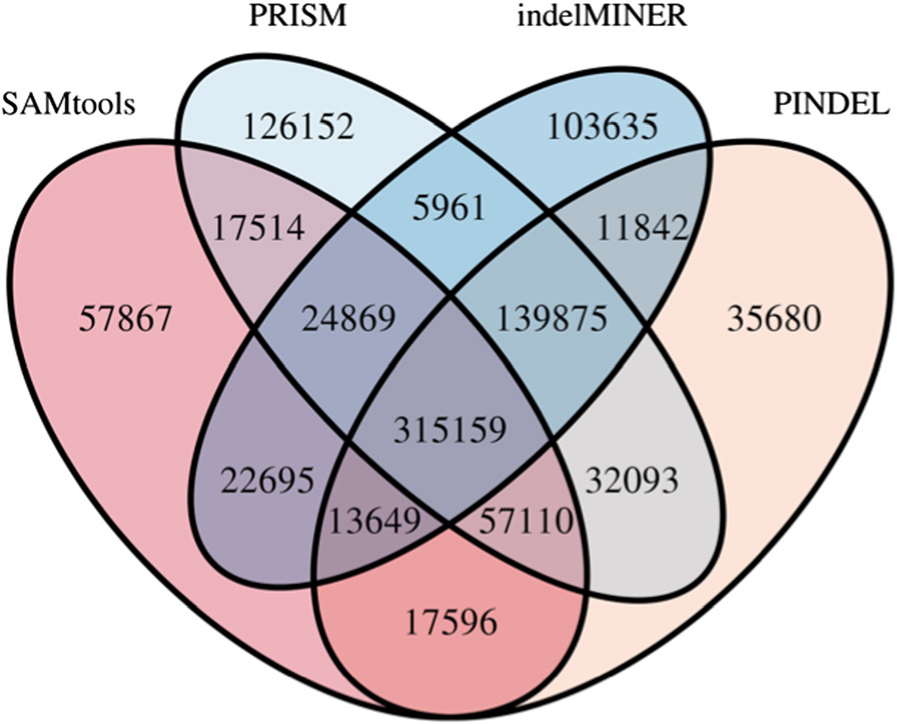
**Comparison of indels identified using SAMtools, PINDEL, PRISM and indelMINER drawn using VennDiagram [** 19 **].**

We also compared the variants identified using indelMINER to those identified using SAMtools, PINDEL and PRISM. Figure 1 shows a comparison of the variants called by the various tools using the same read alignments. Two calls were marked as an overlap if they had a reciprocal overlap greater than 50% of the breakpoint range. All of the included software agreed on 315,159 of the indels, whereas about 658,363 of the indels were supported by at least two of the software we looked at as part of this study.

### Cancer normal/tumor pair

All cancers arise as a result of accumulation of mutations that confer growth advantage. The advent of next-generation sequencing provides a powerful and cost-effective tool to characterize these genome-wide changes. The primary tumor tissue and adjacent or distal normal tissue are frequently sequenced and analyzed to identify germline and rare somatic mutations. The first step in such an analysis is to identify the mutations that are unique to the cancer. Issues such as normal DNA contamination of tumor DNA complicate the analysis by reducing the tumor variant allele frequency.

**Figure 2:**
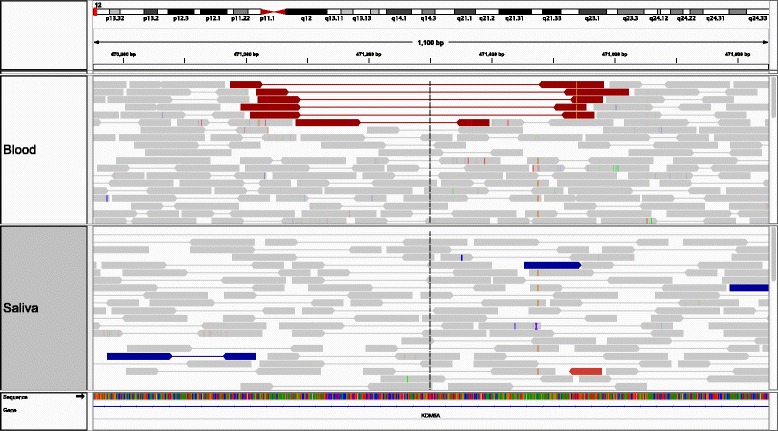
**IGV snapshot of the alignments showing the deletion chr12:471,091-471,494 in the blood sample (top half of the plot) and the absence of the deletion in the saliva sample (bottom half of the plot).**

Large granular lymphocyte (LGL) leukemia is characterized by a clonal expansion of either CD3^+^ cytotoxic T or CD3^−^ NK cells, and is frequently associated with autoimmune diseases such as rheumatoid arthritis [20,21]. A patient was consented under Institutional Review Board protocols initiated at the Pennsylvania State University and continuing at the University of Virginia in accordance with the Declaration of Helsinki. The patient consented to inclusion in an LGL Leukemia patient registry which permits the publication of de-identified patient characteristics and an additional addendum consenting to next generation sequencing and the public deposition of data derived therefrom. We sequenced the peripheral blood and matched saliva from a patient diagnosed with LGL, to a coverage of 29-fold and 17-fold respectively (Additional file 1: Figure S3). We used indelMINER to (a) identify indels in the blood sample and (b) investigate and tag those indels based on their presence or absence in the matched saliva sample. indelMINER identified 575,426 indels in the blood sample, out of which 572,188 of them were also observed in the saliva. Indelocator (https://www.broadinstitute.org/cancer/cga/indelocator) has been used in earlier studies [22] to identify indels in normal/tumor pairs. We used Indelocator on the same dataset, and it identified 478,534 indels in the blood sample, 438,331 of which were also observed in the matching normal sample. We found that 392,512 (82.02%) of the indels found by Indelocator were also found by indelMINER, whereas the remaining indels were observed by only one of the two software tools. We randomly selected 10 indels that were identified by indelMINER but not identified by Indelocator for validation (Additional file 2). We were not able to design a reliable pair of primers for 5 of the indels due to their location in low-complexity regions or repeat regions in the human genome. 4 of the remaining 5 indels were validated using Sanger sequencing, including a large deletion spanning 350 bases (Figure 2).

## Discussion and conclusions

Recent studies have reported on the concordance of single-nucleotide variants identified using different software tools [1] as well using different sequencing platforms [23]. The fraction of polymorphic sites where all platforms/tools agree varies between 70-90% for the SNP calls. Often the overlap of predicted indels between different methods is much lower, indicating that none of the methods offer a comprehensive satisfactory solution.

indelMINER uses a combination of approaches to identify indels of arbitrary size from paired-end short reads. It can predict the exact breakpoint for small and medium size indels, and the approximate breakpoints for the larger deletions. The performance of the algorithm degrades in regions where a single short-read covers multiple indels, as well as in regions where the mapping quality of the sequences is low. A de *novo* assembly approach has been shown to be more suitable in a large fraction of such regions. The current version of indelMINER can only handle indels; however the same algorithm can be extended to handle other types of structural variants, in a manner similar to PINDEL and PRISM. We do not use sequences where both reads from the same fragment align with a mapping quality of zero, i.e., cases where neither of the mates can be aligned unambiguously in finding the indels. If one of the reads can be aligned unambiguously, then indelMINER can use that information to split and align the second read. As explained earlier, we do use such sequences that align ambiguously in the mode where we are just looking to tag the presence or absence of a variant. When tagging the presence or absence of indels in sample B indelMINER uses all the alignments including the secondary alignments to check against the indels found in sample A.

We used both simulated and real data to show that indelMINER has low false-positives and a low false-negative rate when compared to several other tools in the same category. indelMINER can also be used in study of normal/tumor pairs, and in studies where multiple individuals from the same family are being sequenced. The PCR validations confirm the accuracy and sensitivity of indelMINER, and its ability to identify indels in high-throughput sequencing datasets.

## Methods

### Overview

**Figure 3:**
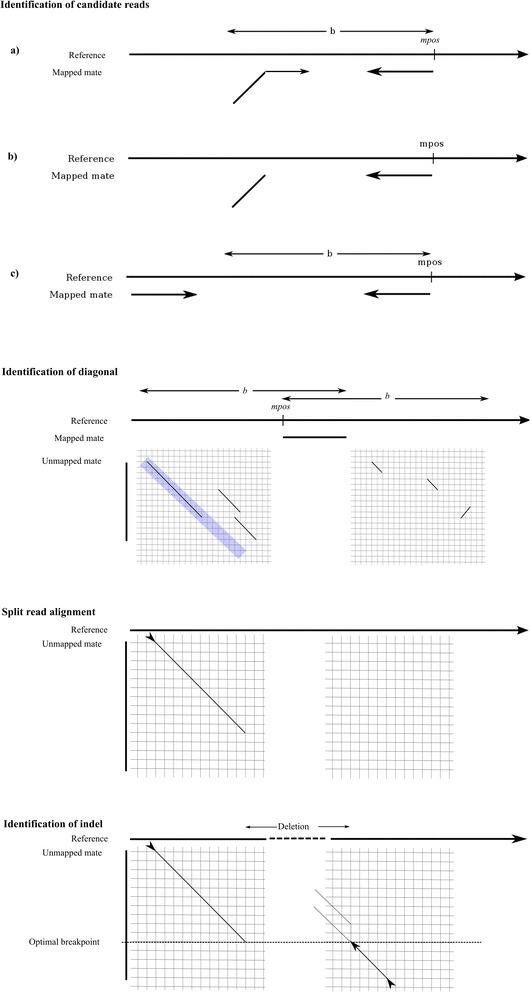
**Overview of the indelMINER algorithm.** Panel titled “Identification of candidate reads” shows three of the cases when a read is identified for realignment or paired-end analysis. **(a)** shows a case when mates align with the expected orientation but one of the mates is only partially aligned, **(b)** shows a case when one mate from a fragment aligns to a location *mpos* on the reference, while the other mate does not align, and **(c)** shows the case when both mates align with the expected relative orientation, but the outer distance constraint is violated. Panel titled “Identification of diagonal” shows the various alignments of the unaligned mate using k-*mer* comparisons, and the subsequent selection of one of the diagonals based on alignment score and distance from *mpos*. Panel “Split read alignment” shows the extension of the chosen diagonal, and the panel “Identification of indel” shows the alignment and extension of the remaining sequence from the unaligned mate, to a region around *mpos* selected based on a user-specified threshold.

indelMINER relies on a combination of split-read and paired-end read approaches to identify indels from a BAM file for a sample (Figure 3). Even though it can be run on any coordinate sorted BAM file, we recommend running the GATK IndelRealigner [10] on it prior to running indelMINER. This local realignment serves to transform regions with misalignments due to indels into clean reads containing a consensus indel that can be then easily identified. The cleaned reads are analyzed in order of their alignments to the reference sequence, and segments of candidate reads are realigned within a specified diagonal band [24], identified using a fast *k-mer* comparison of the read and the reference sequence. These alignments are collected and used to identify candidate insertions and deletions. The identified variants are annotated with additional information pertaining to the region within the breakpoints, including the average depth of coverage, the RMS mapping quality of reads, and the count of reads with a mapping quality equal to zero. These can be used to filter the calls to obtain a more reliable set of differences between the target and the reference genome. Here we describe each of the steps in greater detail.

### Definitions

First we define a few terms that will be used in the description of the workflow and algorithms used in indelMINER.

1. A read group *R (a, b, o)* is defined as a set of paired-end sequences that are the product of a single lane or barcode of a sequencing run. The expected outer distance for the pairs in this group is described by the interval *[a, b]* and the expected relative orientation is given by *o*, where *o ∈ [‘++’, ‘+-’, ‘-+’, ‘--’].* The first symbol represents the orientation of the mate that comes earlier on the chromosomal co-ordinates. For example, the expected relative orientation for Illumina paired-end reads is ‘+-’.
2. A paired-end sequence *P (r1, r2, r, o, i)* consists of two reads *r1* and *r2* that are sequenced from the same DNA fragment. The paired-end fragment belongs to the read group *r*, and *o* and *i* refer to the relative orientation and the outer distance of *r1* and *r2* when both of them are aligned to a reference sequence. The first symbol in *o* defines the orientation of *r1*, and the second symbol defines the orientation of *r2.*
3. *maxsrdelsize* and *maxpedelsize* are user specified thresholds that refer to the maximum size of the deletion that we want to identify using split read and paired-end read approaches respectively.

### Identification of candidate reads

The reads in the BAM file are analyzed in order of their alignment to the reference sequence. A read *r1 (r2)* of a pair *P (r1, r2, r, o, i)* is selected for split read alignment if any of the following conditions is satisfied

- *P (r1, r2, r, o, i)* is properly paired (*o****∈****[‘+-’]* for Illumina PE reads, and *a ≤ i ≤ b* where *r = R (a, b, o))*, and *r1 (r2)* aligns to the reference with one or more indels, or has a unaligned/soft-clipped segment in it. In other words, these are the reads that align to the reference genome with the expected orientation and outer distance, but one of the mates is either aligned partially, or aligned to the reference genome with one or more gaps (Figure 3, Identification of candidate reads (a)).
- The mate *r2 (r1)* is aligned but *r1 (r2)* is unaligned (Figure 3, Identification of candidate reads (b)).

We also collect the pairs *P (r1, r2, r, o, i)* where *r1* and *r2* align to the reference with the expected orientation, but the insert length constraints are not satisfied i.e. *(i < a)* or *(i > b)*, where *r = R (a, b, o)* for a separate paired-end read analysis (Figure 3, Identification of candidate reads (c)).

### Split read alignment of reads

As discussed above, a read *r1* that is selected for split-read analysis has a mate read *r2* that aligns to the reference sequence at a position denoted by *mpos* (Figure 3, Identification of candidate reads). The read *r1* is now aligned to the reference within the interval *[mpos – b, mpos + b],* where *b* refers to the maximum expected outer distance for their read group (Figure 3, Identification of diagonal, Split read alignment). If one end of the read aligns to position *pos* in the above interval, then we attempt to align the read from the other end within the interval *[pos, pos + maxsrdelsize]* or *[pos – maxsrdelsize, pos],* depending on the relative orientation of *r1* and *r2* (Figure 3, Identification of indel). If that fails then we check to see if the unaligned segment of *r1* is a candidate insertion. The above alignments proceed in two steps. First, we use a *k-mer* comparison of the read sequence to the candidate reference segment to find the best diagonal band i.e. the diagonal band where the read and the reference share the most number of unique *k-mers*. The alignments are then performed using a strategy that requires only *O(NW)* computation time and *O(N)* space, where N is the length of the shorter of the two subsequences and *W* is the width of the band [24]. Each split read where both read ends can be aligned in a way that they support an indel, are saved for further analysis.

### Identification of indels

In this step, we collect all the candidate variants *V (e1, e2)*, where *e1* and *e2* refer to either the two split halves as a result of the realignment in the previous step, or refer to the two reads from the same fragment that did not satisfy the outer distance constraints. We create a graph G and represent every candidate variant supported by a split or paired-end read, as a vertex. Two vertices are joined by an edge if they support the same variant in the target genome. If the two vertices represent split-reads, then the only condition for an edge between them is that they support the same breakpoints. If the two vertices represent mates that do not satisfy distance constraints, then an edge can be drawn between them if the resulting breakpoints from the two variants do not violate the outer distance constraint for reads represented by *V1* and *V2*.

Each clique in the graph should now represent a variant in the target genome. However due to errors in sequencing, ambiguous alignments, ploidy, incompleteness and inaccuracies in the reference genome, a significant fraction of these subgraphs are not fully connected. So instead of restricting the definition of a variant to a clique, we consider each connected component in the graph G to represent a variant in the target genome, and the vertices in the connected component to represent the evidence that supports that particular variant. We heuristically identify a maximal clique in each connected component to calculate the putative breakpoints. Since no edges connect split read vertices to the paired read vertices, this analysis can report a single variant as two separate variants, one supported only by split reads, and the other supported only by paired-end reads. The next step in indelMINER involves combining the split read and paired-end read evidence for the same variant. This can be easily accomplished by sorting the variants and then combining the adjacent variants if merging them does not violate the outer distance constraint for the pairs that support the variant.

### Filtering and annotation

The resulting variants can be filtered based on the number of pairs and split-reads that support the variant, the average depth of coverage across the breakpoints, the RMS value of the mapping quality across the breakpoints and the number of reads across the breakpoint that align with a zero mapping quality. We also report the length of the flanking sequences on both sides of the variant for every read that supports the variant. Besides identification, indelMINER can also be used to tag indels with their presence or absence in another sample. This is extremely useful in analysis of normal/tumor pairs and in cases where multiple members of the same family are being analyzed for de-novo variants. This is accomplished by using a list of putative indels in one of the samples “A” (tumor in case of normal/tumor pairs) as input to an instance of indelMINER. indelMINER stores these intervals from sample A, and then processes all improper pairs and split reads in the second sample “B”; including reads with zero mapping qualities, secondary and suboptimal alignments, to find any evidence that can support the breakpoints in “A”. This information is used to annotate the variants as being present or absent in sample B.

## Additional files

Additional file 1:
**The suppmentary methods and figures.**
Additional file 2:
**List of indels chosen for validation and its details.**
