# Supplementary material for: Identification of indels in next-generation sequencing data
Source: BMC Bioinformatics. 2015 Feb 13;16(1):42. doi: 10.1186/s12859-015-0483-6 (PMC4339746; doi:10.1186/s12859-015-0483-6)
Supplement: Additional file 1: — The suppmentary methods and figures. [file 12859_2015_483_MOESM1_ESM.docx]

## SIMULATIONS

We implanted 3,723 known deletions, and 3,777 known insertions [1] into chromosome 22 of the human reference sequence (hg18). The modified sequence was then used as input to simulate 100 bp long, Illumina paired-end reads using pIRS [2]. The reads were generated to an average depth of 20-fold, with insert distances that followed a normal distribution (mean: 500 bps, s.d.: 30 bps).

We aligned the simulated reads to the original chromosome 22 using BWA [3] version 0.5.9. All the default parameters were used and the resulting BAM file was sorted in coordinate order. The reads were realigned around putative indels using IndelRealigner, which is part of the Genome Analysis Toolkit [4] , and putative PCR duplicates were flagged using the MarkDuplicates tool ([http://picard.sourceforge.net](http://picard.sourceforge.net/)). This sorted BAM file was then input to SAMtools [5], PRISM [6], PINDEL [7] and indelMINER and the false-positive rate and the false-negative rate for each of the tools was calculated.

We used the following command line arguments and options with each of the tools:

1. SAMtools : We used the following command line to call the indels using SAMtools

samtools mpileup -C 50 -ugf reference.fa alignments.bam \

| bcftools/bcftools view -bvcgN - \

| bcftools/bcftools view - \

| bcftools/vcfutils.pl varFilter \

| grep "INDEL"

where reference.fa refers to a fasta file which contains the chr22 sequence from hg18, and

alignments.bam refers to the BAM file with the sorted alignments.

1. PINDEL : We used the following command line to call the indels using PINDEL

pindel –f reference.fa -i config.txt -o pindel -x 5

where config.txt contained the information that the expected mean of the insert lengths in the input

BAM file is 500 bps.

1. PRISM : We used the following command line to call the indels using PRISM

samtools sort -n alignments.bam alignments.sorted

samtools view alignments.sorted.bam > alignments.sam

mkdir -p prism_input prism_output

bwasam2prism.py alignments.sam prism_input 500 30

cnver_cluster.sh 500 30 2 prism_input/alignments.sam 100

smapper mapsplit -m 500 -e 30 \

-o prism_output/output \

-S prism_input/alignments.sam_single \

-L prism_input/alignments.sam.links.del.fw \

-l prism_input/alignments.sam.links.del.bw \

-D prism_input/alignments.sam.links.dup.fw \

-d prism_input/alignments.sam.links.dup.bw \

-I prism_input/alignments.sam.links.inver.fw \

-i prism_input/alignments.sam.links.inver.bw \

reference.fa

cat prism_output/output_*.sam >> prism_output/outputall.sam

detect_and_filter.sh \

-i prism_output/outputall.sam \

-r reference.fa

1. indelMINER : We used the command line to call the indels using indelMINER

indelminer –a reference.fa sample=alignments.bam > var.vcf

## NA18507 HUMAN DATA

We ran SAMtools on the resulting BAM file using the following command line:

samtools mpileup -C 50 -Dugf hg19.fa alignments.bam \

| bcftools view -Nvcg - \

| vcfutils.pl varFilter -D 100 \

| grep “INDEL” \

> samtools.out

We ran PINDEL on the BAM file using the following command line:

pindel -f hg19.fa -i config.txt -o pindel.out -x 5

where config.txt is a file with a single line of text

alignments.bam 516 na18507

PRISM was run on the same BAM file using the following commands:

samtools sort -n alignments.bam alignments.sorted

samtools view alignments.sorted.bam > alignments.sam

mkdir chroms

cat alignments.sam | awk '{print $$0 >> "chroms/"$$3".sam"}'

rm alignments.sorted.bam alignments.sam

Then for each chromosome C, the following commands were executed

mkdir -p $C_prism_input $C_prism_output

bwasam2prism.py chroms/$C.sam $C_prism_input 516 45

cnver_cluster.sh 516 45 2 $C_prism_input/$C.sam 100

smapper mapsplit \

-m 516 \

-e 45 \

-o $C_prism_output/output \

-S $C_prism_input/$C.sam_single \

-L $C_prism_input/$C.sam.links.del.fw \

-l $C_prism_input/$C.sam.links.del.bw \

-D $C_prism_input/$C.sam.links.dup.fw \

-d $C_prism_input/$C.sam.links.dup.bw \

-I $C_prism_input/$C.sam.links.inver.fw \

-i $C_prism_input/$C.sam.links.inver.bw \

$R

cat $C_prism_output/output_*.sam >> $C_prism_output/outputall.sam

detect_and_filter.sh \

-i $C_prism_output/outputall.sam \

-r $R

We then ran indelMINER on the resulting BAM file using the following command line:

indelminer -t hg19.fa na18507=alignments.bam > var.vcf

cat var.vcf \

| grep "^#" \

> var.flt.vcf

cat var.vcf \

| grep -v "^#" \

| awk '{

split($8,a,";");

df = int(substr(a[11],4));

ns = int(substr(a[3],4));

dp = int(substr(a[12],4));

nfs = int(substr(a[6],5));

nrs = int(substr(a[7],5));

mq30 = int(substr(a[10],6));

ut = int(substr(a[8],8));

if(df < 4 && dp < 50 && mq30/ns > 0.2 && ut > 2) print $0

}' \

>> var.flt.vcf

The option “-t” instructs indelMINER to ignore the low quality 3’ ends of the reads that were trimmed off by BWA. After the initial calls, we only keep the variants where the average coverage in the region of interest was < 50, and the supporting reads had less than 5 differences (on an average) when compared to the reference genome. We also remove the indels where more than 20% of the reads supporting the variant aligned with a mapping quality < 30.

Figure S1 shows the length distribution of the identified indels for the sample using indelMINER. Figure S2 shows the distribution of these indels across the human chromosomes, which correlate to the amount of DNA in the chromosomes.

Figure S1: Length distribution of the identified indels in NA18507.

Figure S2: The distribution of identified indels for NA18507 across the human chromosomes.

## LGL DATASET

## Study Patient

The patient was a 72 year old female that was consented to a local IRB-approved protocol specific to genetic testing. At the time of sample collection the patient had a five year clinical history of T-cell LGL leukemia with confirmed clonality by T-cell Receptor (TCR) rearrangement testing. The chief symptomatic complaint was chronic neutropenia for which she is occasionally given filgrastim (Neupogen). At the time of collection the patient had an absolute neutrophil count of 600 per microliter. The patient has chronically elevated CD3+/CD8+ T lymphocytes (>85% of lymphocytes, absolute lymphocyte count of 7.6 thousand per microliter). She has not received immunosuppressive therapy, such as methotrexate, for her leukemia.

## Sample Preparation

Saliva and whole blood samples were collected during the same office visit. Saliva was collected with an OGR-500 kit from DNAgenotek and processed according to manufacturer’s protocol. White cells were separated from whole blood by centrifugation over Ficoll-Paque PLUS (GE Health Sciences). DNA was extracted from 5 million cells without further cell purification utilizing a Wizard DNA column (Promega).

## Genome Sequencing and Alignments

The blood and saliva were sequenced using the standard Illumina paired-end library protocol to generated reads of length 101 bps. We aligned the sequences to the human reference (hg19) using BWA [3] version 0.5.9. All the default parameters were used and the resulting BAM file was sorted in coordinate order. The reads were realigned around putative indels using IndelRealigner, and putative PCR duplicates were flagged using the MarkDuplicates tool. Figure S3 shows the depth of coverage distribution in the resulting BAM files for both the blood and the matched saliva sample.

Figure S3: Depth of coverage distribution for the tumor and normal sequences.

## Identification of indels using indelMINER

We ran indelMINER on the BAM file corresponding to the blood sample as follows:

indelminer -i LGL.config -t hg19.fa CS=LGL.bam > LGL.vcf

cat ../CS_LGL.vcf \

| grep -v "^#" \

| awk '{

split($8,a,";");

df = int(substr(a[11],4));

ns = int(substr(a[3],4));

dp = int(substr(a[12],4));

nfs = int(substr(a[6],5));

nrs = int(substr(a[7],5));

mq30 = int(substr(a[10],6));

ut = int(substr(a[8],8));

if(df < 4 && dp < 50 && mq30/ns > 0.2 && ut > 2) print $0

}' \

> LGL.flt.vcf

The option “-t” instructs indelMINER to ignore the low quality 3’ ends of the reads that were trimmed off by BWA. After the initial calls, we only keep the variants where the average coverage in the region of interest was < 50, and the supporting reads had less than 5 differences (on an average) when compared to the reference genome. We also remove the indels where more than 20% of the reads supporting the variant aligned with a mapping quality < 30. The configuration file “LGL.config” contains the minimum and maximum proper insert lengths for all the read-groups in the BAM. These details are calculated by indelMINER if the user does not provide them, but that can increase the run time significantly. The contents of the configuration file in this command line are as follows:

IL CS_LGL_2 20 500

IL CS_LGL_4 31 500

IL CS_LGL_1 23 500

IL CS_LGL_3 35 500

CS_LGL_1, CS_LGL_2, CS_LGL_3, and CS_LGL_4 refer to the 4 read-groups in the BAM file, and each line begins with “IL” to specify that the corresponding lines refer to insert-length details.

indelminer \

-i Saliva.config \

-n 0 -q 0 -t hg19.fa LGL.flt.vcf CS_saliva=Saliva.bam \

> LGL.flt.tag.vcf

This command tags the variants in LGL.flt.vcf based on their absence or presence in the BAM file called Saliva.bam. The options “-n 0 –q 0” instruct indelminer to consider all reads (by default, only reads with a mapping quality >= 10 are considered) and all bases in the reads (by defaults indels are not called in the last 10 bps of the reads). The contents of the configuration file used in this command is as follows:

IL CS_Saliva_7 40 450

IL CS_Saliva_5 47 450

IL CS_Saliva_8 35 450

IL CS_Saliva_6 46 450

## Identification of indels using Indelocator

We ran Indelocator (<https://www.broadinstitute.org/cancer/cga/indelocator>) on the same LGL dataset to identify indels as follows:

java -Xmx10g \

-jar IndelGenotyper.36.3336-GenomeAnalysisTK.jar \

--analysis_type IndelGenotyperV2 \

--somatic \

--reference_sequence hg19.fa \

--input_file:normal Saliva.bam \

--input_file:tumor LGL.bam \

--out LGL.indelocator.vcf

## Validation of indels

Primers were designed to the 250 basepair flanking regions on either side of each called indels (hg19, UCSC genome browser). Primers were designed with a target amplicon size of 400-500 basepairs. MyTaq Red (Bioline) was used in 50 ul reactions for 35 cycles to generate products using 50 nanograms of genomic DNA from saliva or blood sample per reaction. Large indels were visualized on a 1.8% agarose gel stained with Sybr Safe (Life Technologies) on a ChemiDoc XRS (Bio-Rad). PCR products for predicted small indels were extracted with Chargeswitch columns (Life Technologies) and sequenced in the UVa DNA Sciences Core on the Applied Biosystems 3730 DNA Analyzer using BigDye Terminator 3.1 chemistry. The forward primer from each PCR was used for sequencing. Sequences were viewed in Sequencher 5.1 (Gene Codes). The details of the primers used for the validations are available in the Excel spreadsheet “Supplementary Data.xlsx”.

## REFERENCES

1. Levy S, Sutton G, Ng PC, Feuk L, Halpern AL, Walenz BP, Axelrod N, Huang J, Kirkness EF, Denisov G, Lin Y, MacDonald JR, Pang AWC, Shago M, Stockwell TB, Tsiamouri A, Bafna V, Bansal V, Kravitz S a, Busam D a, Beeson KY, McIntosh TC, Remington K a, Abril JF, Gill J, Borman J, Rogers Y-H, Frazier ME, Scherer SW, Strausberg RL, et al.: **The diploid genome sequence of an individual human.** *PLoS Biol* 2007, **5**:e254.

2. Hu X, Yuan J, Shi Y, Lu J, Liu B, Li Z, Chen Y, Mu D, Zhang H, Li N, Yue Z, Bai F, Li H, Fan W: **pIRS: Profile-based Illumina pair-end reads simulator.** *Bioinformatics* 2012, **28**:1533–5.

3. Li H, Durbin R: **Fast and accurate short read alignment with Burrows-Wheeler transform.** *Bioinformatics* 2009, **25**:1754–60.

4. McKenna A, Hanna M, Banks E, Sivachenko A, Cibulskis K, Kernytsky A, Garimella K, Altshuler D, Gabriel S, Daly M, DePristo M a: **The Genome Analysis Toolkit: a MapReduce framework for analyzing next-generation DNA sequencing data.** *Genome Res* 2010, **20**:1297–303.

5. Li H, Handsaker B, Wysoker A, Fennell T, Ruan J, Homer N, Marth G, Abecasis G, Durbin R: **The Sequence Alignment/Map format and SAMtools.** *Bioinformatics* 2009, **25**:2078–9.

6. Jiang Y, Wang Y, Brudno M: **PRISM: Pair read informed split read mapping for base-pair level detection of insertion, deletion and structural variants.** *Bioinformatics* 2012:1–7.

7. Ye K, Schulz MH, Long Q, Apweiler R, Ning Z: **Pindel: a pattern growth approach to detect break points of large deletions and medium sized insertions from paired-end short reads.** *Bioinformatics* 2009, **25**:2865–71.
